# Immunization with Pre-Erythrocytic Antigen CelTOS from *Plasmodium falciparum* Elicits Cross-Species Protection against Heterologous Challenge with *Plasmodium berghei*

**DOI:** 10.1371/journal.pone.0012294

**Published:** 2010-08-19

**Authors:** Elke S Bergmann-Leitner, Ryan M. Mease, Patricia De La Vega, Tatyana Savranskaya, Mark Polhemus, Christian Ockenhouse, Evelina Angov

**Affiliations:** 1 Division of Malaria Vaccine Development, United States Military Malaria Vaccine Program, Walter Reed Army Institute of Research, Silver Spring, Maryland, United States of America; 2 Division of Entomology, Walter Reed Army Institute of Research, Silver Spring, Maryland, United States of America; Nuffield Orthopaedic Centre, United Kingdom

## Abstract

**Background:**

The *Plasmodium* protein Cell-traversal protein for ookinetes and sporozoites (CelTOS) plays an important role in cell traversal of host cells in both, mosquito and vertebrates, and is required for successful malaria infections. CelTOS is highly conserved among the *Plasmodium* species, suggesting an important functional role across all species. Therefore, targeting the immune response to this highly conserved protein and thus potentially interfering with its biological function may result in protection against infection even by heterologous species of *Plasmodium*.

**Methodology/Principal Findings:**

To test this hypothesis, we developed a recombinant codon-harmonized *P. falciparum* CelTOS protein that can be produced to high yields in the *E. coli* expression system. Inbred Balb/c and outbred CD-1 mice were immunized with various doses of the recombinant protein adjuvanted with Montanide ISA 720 and characterized using *in vitro* and *in vivo* analyses.

**Conclusions/Significance:**

Immunization with PfCelTOS resulted in potent humoral and cellular immune responses and most importantly induced sterile protection against a heterologous challenge with *P. berghei* sporozoites in a proportion of both inbred and outbred mice. The biological activity of CelTOS-specific antibodies against the malaria parasite is likely linked to the impairment of sporozoite motility and hepatocyte infectivity. The results underscore the potential of this antigen as a pre-erythrocytic vaccine candidate and demonstrate for the first time a malaria vaccine that is cross-protective between species.

## Introduction

Malaria remains a significant disease in most tropical countries and despite prophylactic efforts in the form of bed nets and the development of novel anti-malarial drugs, the disease is still a major threat to global health and the survival of children under the age of five. The current lead malaria vaccine, now in Phase III trials, GlaxoSmithKline's RTS,S vaccine, has shown a 53% reduction in clinical episodes of malaria for eight months in children 5 to 17 months old [1]; and in a trial in Mozambique children aged 1 to 4 years, the vaccine was capable of providing protection for up to 45 months, but at a lower efficacy [2]. Although the results from RTS,S vaccination are encouraging, there remains a need to increase the observed vaccine efficacy of pre-erythrocytic vaccine candidates and this may be achieved by either modifying the adjuvant partners, changing the vaccine delivery platform or by the addition of new antigens. These approaches have primarily focused on the circumsporozoite protein (CSP) (reviewed in [3]) as the target antigen and as such it is not clear whether other pre-erythrocytic antigens can substitute for CSP or can act in combination with a CSP-based vaccine to achieve the required increase in vaccine efficacy. Moreover, immunity based on anti-CSP responses is species-specific [4], [5] and thus the CS proteins of the different *Plasmodium* species would have to be included in a combination vaccine approach in order to protect individuals living in areas with mixed *Plasmodium* species.

Genomic and proteomic data mining have identified novel *Plasmodium* antigens that are either restricted to the sporozoite and/or the early liver-stages of the mammalian life cycle. One of these antigens is the cell-traversal protein for ookinetes and sporozoites (CelTOS) identified independently by two laboratories through either comparative screening of genomic databases for immunogenic antigens [6] or using suppression subtractive hybridization (SSH) of *Plasmodium yoelii* sporozoites versus merozoites [7]. Shortly thereafter, the antigen was identified again by screening *P. berghei* expressed sequence tag (EST) databases of salivary glands and ookinetes [8]. Using *P. berghei* as the model, genetic disruptions of *celtos* not only reduced sporozoite infectivity of the liver, but also reduced cell passage through the liver sinusoid as well as reduced passage through the midgut epithelium by ookinetes [8]. The notion that CelTOS is an important protein for the traversal of the malaria parasite in both, the mammalian and the insect host, warranted an evaluation of whether targeted immune responses against this antigen could prevent the infection of the liver in the mammalian host. Concomitantly, others have attributed immunological significance to CelTOS by demonstrating that CelTOS-specific peptides can stimulate the PBMC's isolated from volunteers immunized with irradiated sporozoites, a highly effective vaccine inducing sterile protection, to produce antigen-specific IFN-γ responses, emphasizing the potential immunological impact of this antigen [6]. This study reported that CelTOS-specific peptides stimulate the highest IFN-γ responses in PBMC's, higher than even using CSP or, TRAP, LSA1 and EXP1 or any of the other newly discovered antigens being evaluated. CelTOS may also be an attractive target since the protein is highly conserved [8] thus potentially requiring only a single subunit vaccine to induce broadly protective immunity against multiple *Plasmodium* species.

The objective of the present study was to evaluate the capacity of the recombinant CelTOS protein to induce sterile protection in mice against a heterologous challenge with *P. berghei* sporozoites. First, we codon-harmonized the gene sequence of PfCelTOS for optimal expression in *E. coli* based on our previously published algorithm [9]. This resulted in high expression levels of a soluble, full length protein. Various doses of the CelTOS protein emulsified with Montanide ISA-720 were tested for their protective potential after three immunizations. Optimal antigen doses consistently induced sterile protection in 50–60% of both, outbred as well as inbred mice. Characterization of the immune response revealed that both humoral and cellular responses may play a role in the heterologous protection against *P. berghei* challenge. Currently, the immune mechanism(s) correlating with protection mediated by CelTOS remain to be elucidated. These results are quite encouraging as they represent the first vaccine approach mediating protection against a heterologous sporozoite challenge.

## Results

### Immunization with PfCelTOS results in sterile protection against heterologous challenge with *P. berghei* sporozoites in inbred and outbred mice

**Figure 1:**
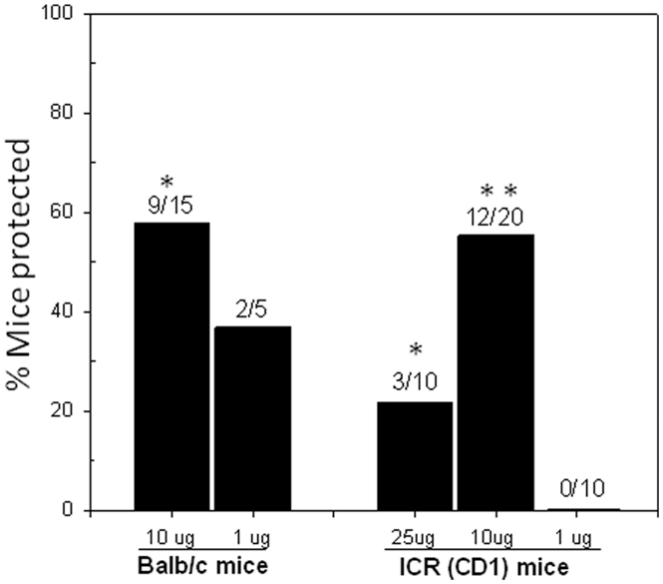
Immunization with recombinant PfCelTOS induces sterile protection against a heterologous sporozoite challenge with *P. berghei*. Inbred Balb/c mice and outbred CD-1 mice were immunized three times with the indicated doses and challenged two weeks after the last immunizations by subcutaneous injection of either 4,000 (for Balb/c) or 15,000 (for CD-1 mice) sporozoites. Vaccine efficacy was calculated using the formula: Efficacy  =  [1-[(number of infected animals (I)_vaccine_/total number of animals (n)_vaccine_)÷(number of infected animals _control_ (I)/total number of animals (n) _control_)]]*100. The numbers above bars represent the number of protected animals/total number of animals in the group. Statistical significance was determined with Fisher Exact test (two-tailed) and is indicated by asterisks (* p = 0.01, ** p = 0.001).

To determine whether CelTOS is a protective antigen against heterologous challenge, Balb/c and CD-1 mice were immunized with different doses of *P. falciparum* CelTOS recombinant protein (PfCelTOS) (Purification profile Figure S1) adjuvanted with Montanide ISA 720 and challenged subcutaneously with *P. berghei* sporozoites two weeks after the last immunization. Challenge control mice received saline adjuvanted with Montanide ISA 720. Mice that did not develop parasitemia by day 14 were considered to be protected (Figure 1). PfCelTOS induced protection in both, inbred and outbred animals that was statistically significant compared to control Montanide ISA 720 for Balb/c mice using the 10 µg dose (p = 0.01 Fisher's exact T-test) and for CD-1 mice using both the 25 µg dose (p = 0.01) and the 10 µg dose (p = 0.001). However, the optimal dose was 10 µg protein/immunization emulsified with Montanide ISA 720 for both strains of mice achieving sterile protection in approximately 60% of the mice. Interestingly, the dose response in CD-1 mice was not linear, with the higher dose tested, 25 µg, resulting in reduced efficacy compared with the 10 µg dose.

### Immunization with PfCelTOS adjuvanted in Montanide ISA 720 results in high antibody titers

**Figure 2:**
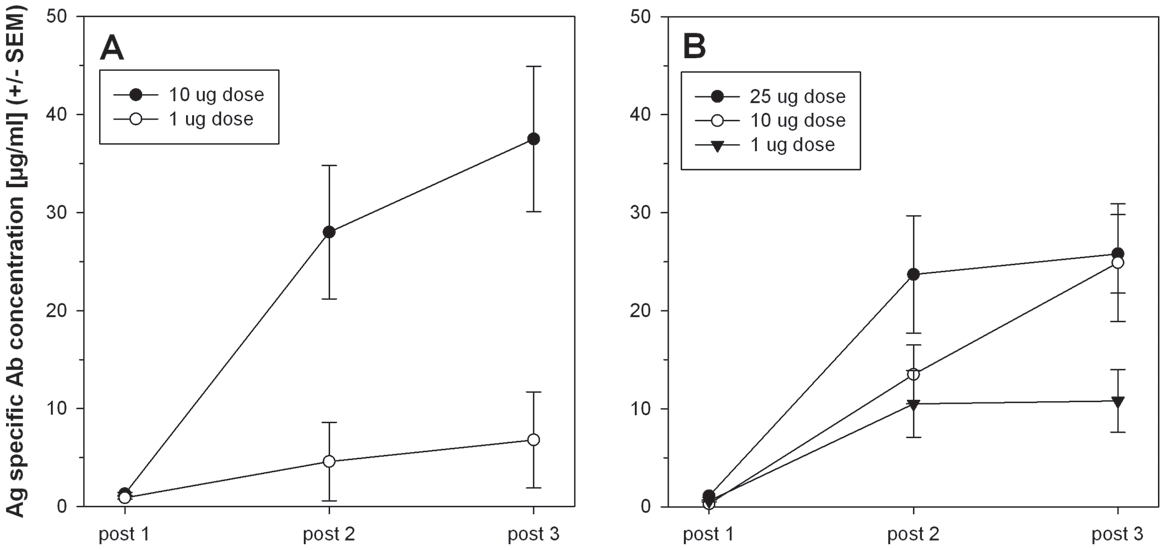
PfCelTOS induces a strong humoral immune response in inbred and outbred mice. Sere were collected three weeks after the first and second immunization (post-1, post-2) and two days prior to challenge (post-3). Antibody kinetics for Balb/c mice (Panel A) and CD-1 mice (Panel B) were determined using a quantitative ELISA. Data are expressed as the mean µg/ml, error bars represent the SEM of n = 15 mice/group (i.e., 10 challenged mice and 5 reagent mice/group).

**Figure 3:**
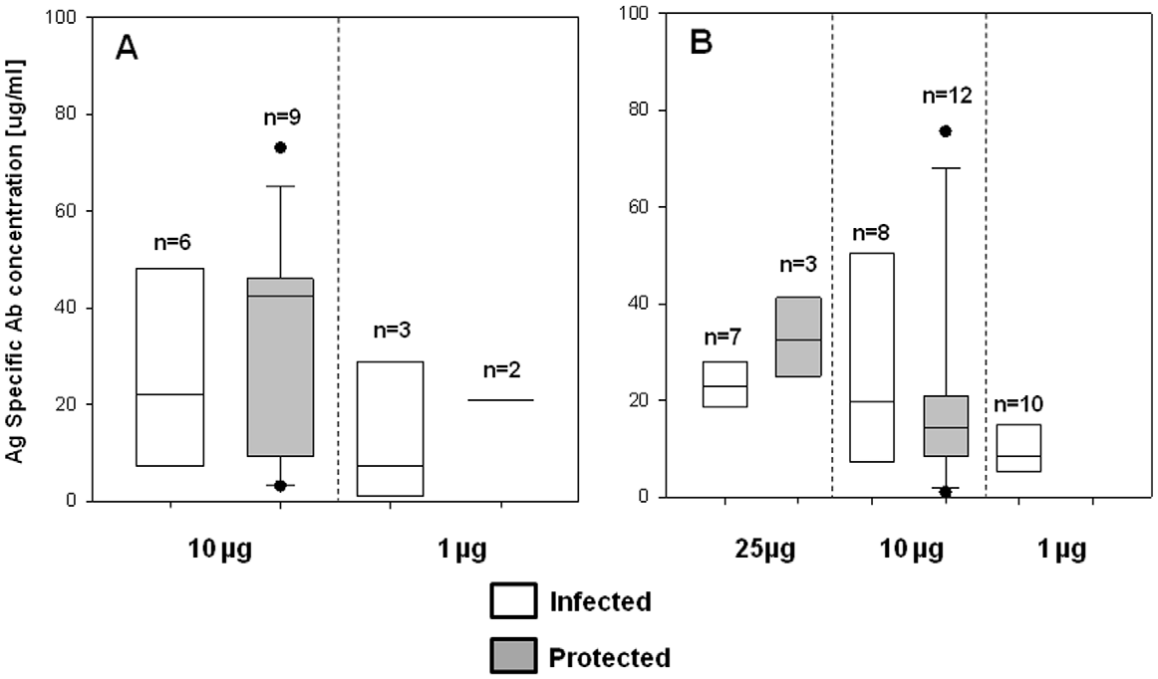
PfCelTOS specific antibody concentration does not predict protective status of mice. Data of pre-challenge sera from all tested dose groups were stratified by protective status of the animals. Box plot indicates the median and the boundaries indicate the 25^th^ and the 75^th^ percentile.

To evaluate the immunogenicity of PfCelTOS, inbred Balb/c and outbred CD-1 mice were immunized three times with various doses of recombinant PfCelTOS emulsified in Montanide ISA 720. Sera were collected two days prior to each immunization (post 1, post 2) or 2 days prior to challenge (post 3) and tested in a quantitative ELISA (Figure 2). The antibody response in both strains reached a saturation plateau after the second immunization when immunized with the highest vaccine dose tested. As the mechanism of protective immunity by this vaccine is not known, we stratified the pre-challenge antibody-data by protective status of the animals (Figure 3). While there was a trend towards higher medians in some of the protected animals, there was no statistical difference between mice that later became infected versus the mice that were protected after challenge.

### CelTOS-specific monoclonal and polyclonal antibodies react with sporozoites

**Figure 4:**
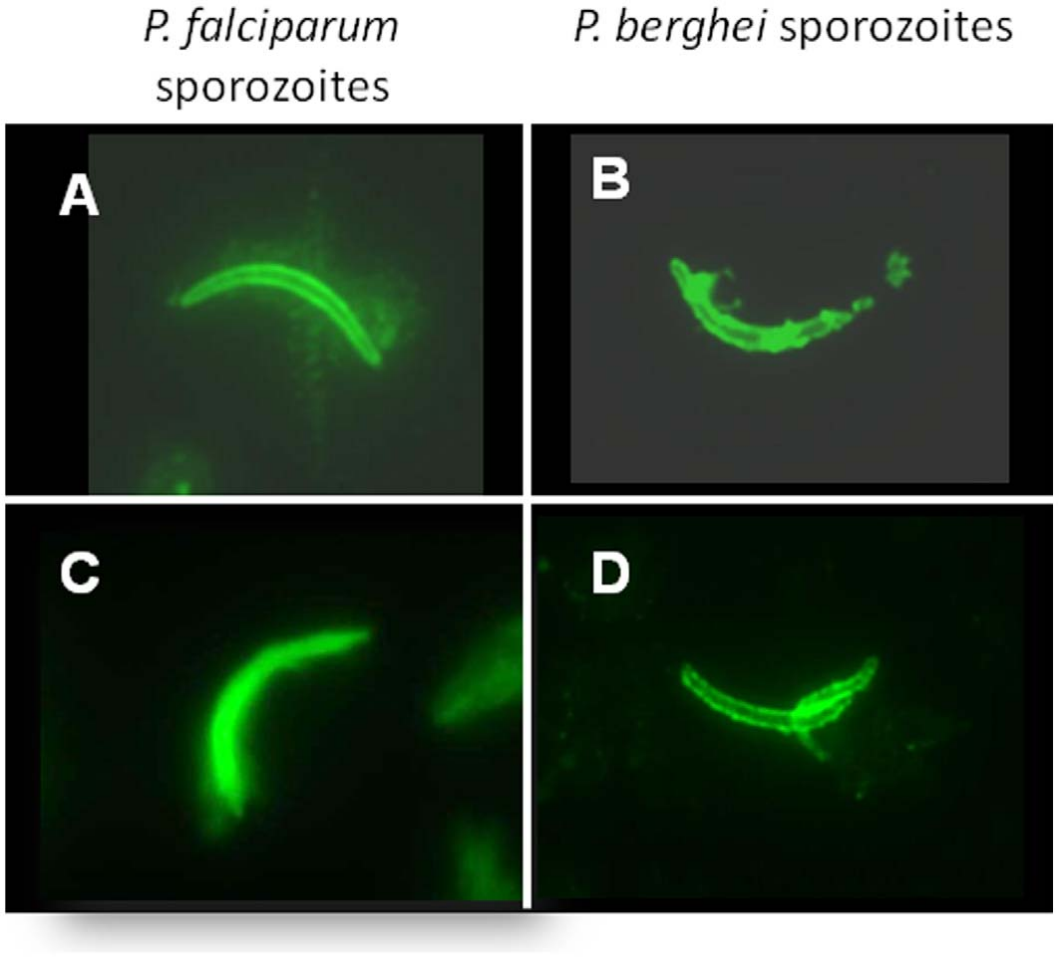
PfCelTOS-specific antibodies raised by protein immunization react with *P. falciparum* and *P. berghei* sporozoites. Reactivity of polyclonal anti-PfCelTOS antibodies against *P. falciparum* (Panel A) and *P. berghei* sporozoites (Panel B) and reactivity of PfCelTOS-specific mAb 2C7 against *P. falciparum* (Panel C) and *P. berghei-*specific mAb 15E2 (Panel D) against *P. berghei* sporozoites. The antisera used for the staining were from the post-3 sera ( =  prechallenge) and were tested at 1∶200 dilutions. Images were taken at 1,000x magnification.

We next sought to determine whether antibodies induced by recombinant protein immunization could recognize native antigen on or inside dissected salivary gland sporozoites (Figure 4). Immunostaining using polyclonal anti-PfCelTOS antisera from Balb/c mice immunized with PfCelTOS/ISA 720 (post-3 sera) showed reactivity with homologous *P. falciparum* (Panel A) as well as with the heterologous *P. berghei* sporozoites (Panel B). Positive control staining of *P. falciparum* sporozoites (Panel C) and *P. berghei* sporozoites (Panel D) was performed using PfCelTOS-specific mAb 2C7 or PbCelTOS-specific mAb 15E2, respectively (produced in our laboratory). Sera from mice that had only received the adjuvant, as well as pre-immune sera, did not react with the sporozoites (data not shown).

### CelTOS-specific antibodies inhibit invasion of hepatocytes by *P. falciparum* sporozoites *in vitro*

**Figure 5:**
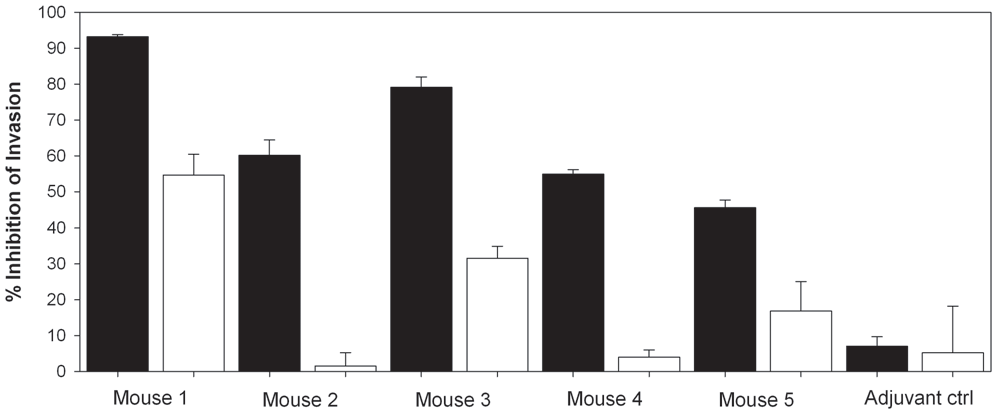
CelTOS-specific antibodies inhibit invasion of hepatocytes by *P. falciparum* sporozoites. Sera from mice immunized either with CelTOS (and protected against challenge) or adjuvant alone were tested in triplicate for their ability to inhibit hepatocyte infection *in vitro*. HepG2A16 cells were incubated with sporozoites in the presence or absence of antisera at either 1∶100 (black bars) or 1∶400 (white bars). Percent inhibition was calculated based on the number of infected hepatocytes in the control cultures.

Gene knockout experiments suggest that CelTOS may be an advantageous immunological target against hepatocyte infectivity (8). Thus we designed experiments to determine whether antibodies directed against the antigen would impair the ability of sporozoites to invade hepatocytes *in vitro*. Antibodies from mice immunized with CelTOS adjuvanted with Montanide ISA-720 inhibited invasion in a dose-dependent manner while sera from mice immunized with only adjuvant did not inhibit the invasion (Figure 5).

### CelTOS-specific antibodies can inhibit the motility of *P. falciparum* sporozoites *in vitro*

It has been reported that live sporozoites deposit a trail of CSP on glass [10]. As sporozoite motility is an indirect measure of the viability and health of the parasites, we chose the motility assay as a functional readout to determine whether antibodies to CelTOS could inhibit the motility of sporozoites and thus could provide an explanation for how invasion inhibition was mediated. To this end, mature *P. falciparum* sporozoites (day 18–20) from salivary gland dissection were incubated for 15 min with either control serum or PfCelTOS-specific antisera or an anti-PfCSP specific monoclonal antibody (clone 216.5G10) (Figure S2). After the pre-incubation, the sporozoites were plated without washing onto glass slides and incubated for 1 hr at 37°C. Incubation of sporozoites with control mouse serum (either pre-immune or serum from adjuvant alone injected mice) resulted in the deposition of CSP trails on slides thus establishing the overall fitness of the sporozoites (Panel A). Similarly, incubation of sporozoites with CSP-specific mAbs resulted in extensive trails that often were more pronounced and stronger than the ones found in the control slides (Panel B) [11], [12]. In contrast, the incubation of sporozoites with anti-PfCelTOS specific antisera led to either reduced trails or no trails at all (Panel C). A quantitative analysis of the number of trails observed in the presence or absence of PfCelTOS-specific antisera is shown in Table 1 demonstrating that CelTOS antibodies have the ability to interact with and/or interfere with the apparent motility of sporozoites. In two of the three experiments we observed an 85% reduction in the total number of trails (p = 0.009 two sample T-test comparing number of trails in control vs. number of trails in the CelTOS group). Moreover, when testing the PfCelTOS-specific antibodies and their effect on heterologous *P. berghei* sporozoites, we observed a similar reduction in total number of trails and motility of sporozoites (data not shown).

**Table 1:** Quantitative analysis of the effect of anti-PfCelTOS specific antibodies on the motility of *P.falciparum* sporozoites.

	Control seruma,b		PfCelTOS specific serum^b^		PfCSP specific mAb^c^	
	n spz	n trails	n spz	n trails	n spz	n trails
Expt 1	46	28	40	8	41	30
Expt 2	130	96	112	9	109	60
Expt 3	62	26	48	3	61	35

a Serum from mice immunized with saline/Montanide ISA 720.

b Serum concentration was 5% (vol/vol) during the 15 min pre-incubation.

c Concentration of mAb 5G10  = 50 µg/ml.

### Immunization with PfCelTOS adjuvanted in Montanide ISA 720 induces IFN-γ as well as IL-4 producing T cells reactive to homologous and heterologous protein

**Figure 6:**
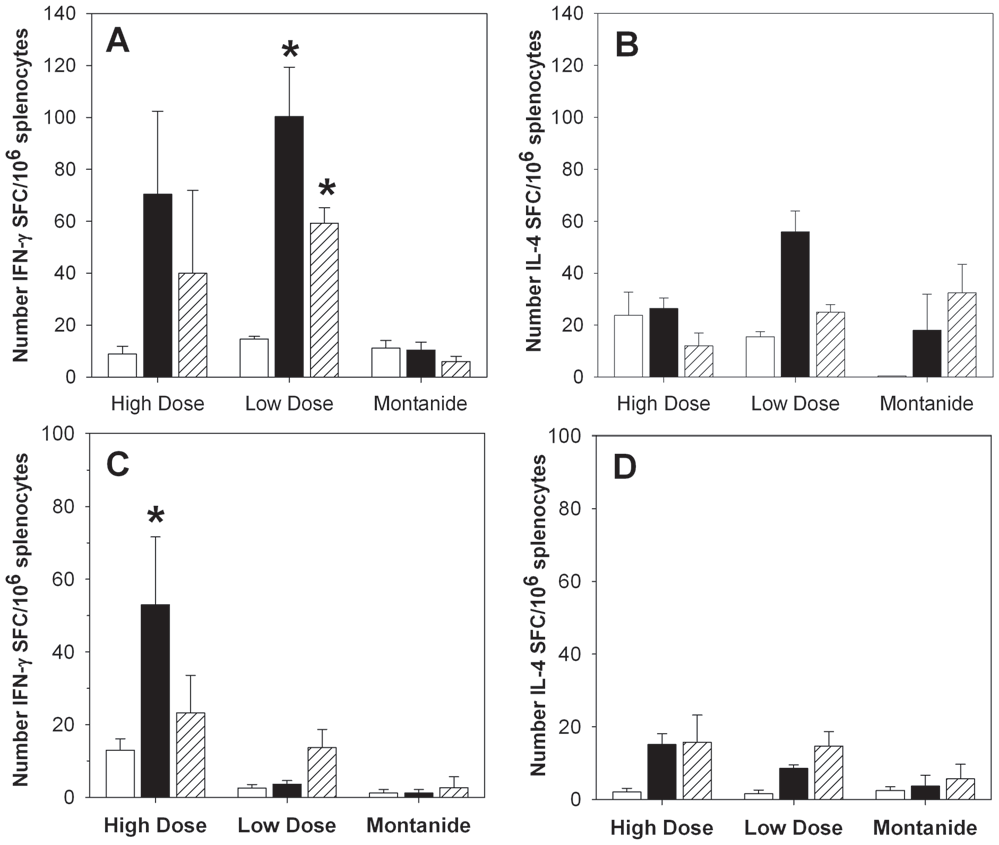
PfCelTOS specific IFN-γ and IL-4 responses show significant cross-reactivity to the heterologous protein. Splenocytes from Balb/c mice (Panel A, B) and CD-1 mice (Panel C, D) immunized three times with recombinant protein in Montanide ISA 720 were tested in IFN-γ (Panel A,C) and IL-4 (Panel B,D) specific ELISpot assays. Data are expressed as the mean number plus standard error of the mean (SEM) of cytokine producing cells (spot forming cells, SFC)/10^6^ splenocytes of individual mice (n = 5/group). Immunization doses for Balb/c  = 10 µg (high dose), 1 µg (low dose), immunization dose for CD-1  = 25 µg (high dose), 10 µg (low dose). Antigens used for *ex vivo* stimulation: no antigen (white bar), 10 µg/ml PfCelTOS (black bar), 10 µg/ml PbCelTOS (hatched bar). For the statistical analysis, vaccinated groups were compared with the Montanide ISA 720 control group stimulated with the same antigen (* indicates p<0.05, two-sided T-test).

As no immune correlate has been established for this malaria vaccine, we sought to characterize the humoral and the cellular immune response. To this end, the frequencies of CelTOS-specific IFN-γ or IL-4 producing T cells in spleens from inbred Balb/c and outbred CD-1 mice were measured using an established ELISpot assay (Figure 6). This analysis yielded several key findings: Firstly, in Balb/c mice PfCelTOS-specific splenocytes showed a contemporaneous IL-4 and IFN-γ response while splenocytes from CD-1 mice produced primarily IFN-γ indicating a bias towards Th1. Secondly, a higher number of PfCelTOS-specific T cells were induced compared to the higher dose tested in Balb/c. Thirdly, the cellular responses in CD-1 mice were lower overall compared to responses in Balb/c mice. Lastly, cross-reactive T cell responses were observed in both mouse strains when splenocytes were stimulated with the heterologous antigen, PbCelTOS.

## Discussion

CelTOS has been shown to play a pivotal role in the cell traversal of host cells by malaria parasites [8]. The biological function for this antigen was described using knockout parasites that showed a significant reduction in infectivity of cells in both, the mosquito host as well as the mammalian host [8].

The present study is the first demonstration of the immunological impact of an immune response directed against CelTOS. First, we generated a recombinant PfCelTOS in the *E. coli* expression system using the codon harmonized gene sequence from *P. falciparum*. The CelTOS protein is highly conserved with 67% similarity at the amino acid level between the two *Plasmodium* species relevant to this analysis, *i.e. P. falciparum* and *P. berghei*. Based on the genetic conservation, *celtos* may belong to the category of malaria genes that have evolved in a common ancestor of *Plasmodium* parasites. Sequence analysis of 92 blood samples collected from around the world resulted in 98% homology among all *P. vivax* and 91% homology among all isolates tested [13]. The apparent lack of genetic diversity and antigenic polymorphism which has commonly thwarted the development paths for specific malaria vaccine candidates, such as AMA1, MSP1 and MSP2 [14], for example, warrants the further evaluation of CelTOS as a protective antigen.

The principal finding from this study was the demonstrated protective efficacy of an anti-PfCelTOS immune response against sporozoite challenge with a heterologous species, *P. berghei* a phenomenon not previously observed with any other malaria antigen. Using this protein as a vaccine, we conclude that CelTOS is a protective antigen conferring sterile immunity in a proportion of both inbred Balb/c and outbred CD-1 mice (Figure 1). Interestingly, a apparent inverse correlation between immunizing dose and vaccine efficacy was observed for both mouse strains with the 10 µg cohorts having the highest vaccine efficacy against the heterologous challenge. We observed that increasing the dose for the immunization of CD-1 mice resulted in a reduction of the protection while the humoral and the cellular responses showed similar or higher responses at that vaccine dose. It may be that the higher dose resulted in the loss of high-affinity clones of either B or T cells which are likely the biologically relevant effectors [15], [16]. The apparent efficacy of this vaccine approach may be further increased by either testing the PfCelTOS in a homologous challenge model (e.g., either transgenic *P. berghei* parasites expressing PfCelTOS) and/or by modifying the adjuvant system or immunization regimen. Preliminary data suggest that epidermal plasmid-based immunization for PfCelTOS by gene gun did not improve vaccine efficacy when the DNA-immunized mice were challenged with sporozoites (Bergmann-Leitner et al., manuscript in preparation). These results were surprising as this vaccine platform was shown to be an effective delivery route for the *P. berghei* CSP vaccine developed in our laboratory [17], [18], [19]. We are currently evaluating other vaccine platforms and adjuvant formulations using this antigen.

The analysis of the vaccine-induced immune responses revealed that both arms of the adaptive immune system are being activated. The serological response in Balb/c mice was significantly higher than the CelTOS-specific antibody concentrations measured in CD-1 mice (Figure 2, 3), but this is not unexpected since inbred mice will have a more homogenous response and - depending on the MHC allele - the magnitude of the responses induced may differ. When analyzing the kinetics of the humoral immune response induced by PfCelTOS (Figure 2) it became apparent that two immunizations may suffice to achieve high antibody titers in Balb/c mice. The serological response in CD-1 mice was lower compared to the response observed in Balb/c mice and may indicate genetic restriction where more immunogenic epitopes bind to H2d.

Stratifying the serological data based on the protective status failed to identify antibody as the immune correlate of protection (Figure 3). This does not exclude antibodies for being the mediator of protection as it may not be the magnitude but rather the quality (such as affinity) or the fine specificity of the humoral response. Similar failure in correlating total antibody levels with protection has been documented for other malaria antigens as well. For example, evaluating MSP-1p42 as protective antigen in *Aotus* monkeys failed to show correlation between total antibody titers and protection against challenge with bloodstage parasites [20] and a study employing C3d as molecular adjuvant for the circumsporozoite protein resulted in high antibody titers, but reduction in protective efficacy. Further investigation determined that shifts in the fine specificities of the antibody response that was not detectable by ELISA using the full length protein was responsible for the loss of protection [21]. We are currently exploring in depth the contribution of the various arms of the immune system in CelTOS-mediated protection.

The antibodies induced by recombinant CelTOS protein were able to bind to native CelTOS expressed by the sporozoites and were able to inhibit the invasion of sporozoites into HepG2 hepatoma cells. The most significant finding in this study with respect to a possible mechanism by which a humoral anti-CelTOS response would not only inhibit the invasion of sporozoites into hepatocytes, but also confer protection, came from the *in vitro* hepatocyte invasion inhibition assay (ISI) (Figure 5) and the sporozoite motility assays (Figure S2). Sera from CelTOS-immunized and protected animals were able to inhibit hepatocyte invasion in a dose dependent manner, while sera from adjuvant control mice were not. Furthermore, motility assays were performed to characterize whether CelTOS-specific antibodies were able to act against sporozoites prior to reaching their final target. While control mouse antibodies or anti-CSP specific antibodies had no effect on the motility of sporozoites (Figure S2A, B), anti-CelTOS specific antibodies greatly inhibited their motility as measured by the deposition of CSP onto the glass slides (Figure S2C). These results suggest that antibodies targeted to CelTOS, a secreted protein, impact on sporozoite motility, at least *in vitro*, through their ability to inhibit the shedding of CS protein from the sporozoite. The exact nature of the mechanism by which these antibodies may impair/abrogate the sporozoites motility remains to be determined.

The profile of the cellular immune response induced by the PfCelTOS protein was investigated in both inbred Balb/c mice and outbred CD-1 mice (Figure 6). Similar to the observation made for the humoral immune response, we found that the vaccine induced higher number of CelTOS-specific IFN-γ and IL-4 producing splenocytes in Balb/c than in CD-1 mice. Moreover, CD-1 mice had only a marginal number of total IL-4 producing cells compared to control Montanide ISA 720 immunized mice. Splenocytes of both mouse strains were also able to recognize the heterologous CelTOS protein from *P. berghei* indicating cross-reactivity at the cellular level.

Taken together, these data suggest that immunization with the conserved sequence of the CelTOS protein leads to significant cross-reactivity at both the humoral and cellular level and to significant cross-protection from a heterologous challenge. While this is the first such reporting of CelTOS as a protective antigen, we believe that further optimization by changing either the regimen and/or adjuvant system or even the vaccine delivery platform may lead to even higher protective responses, especially once the antigen can be tested in the appropriate homologous challenge model.

Although the aim of this study was to assess protective efficacy against sporozoite challenge, an alternate line of investigation for CelTOS-specific antibodies based on the natural role of the CelTOS antigen in cell traversal by ookinetes in addition to that of sporozoites, is at the sexual blood-stage development within the mosquito host. We are currently evaluating the transmission blocking capability of CelTOS-specific antibodies to act against ookinetes. The mode of action of these CelTOS-specific antibodies would be through binding on ookinetes and blocking their traversal through the midgut and thus abrogating further development to oocysts in the basal lamina. Although highly speculative, if antibodies to CelTOS were to have such long ranging activities against both the mammalian and mosquito hosts, significant strides toward malaria eradication could be envisioned. This study represents the first such demonstration of protective efficacy and induction of anti-CelTOS antibodies functional against the sporozoite stage of the *P. falciparum*. These results are encouraging and support further investigations into CelTOS-based vaccine approaches and the immune mechanisms leading to the observed protective effect.

## Materials and Methods

### Ethics Statement

The HepG2, human cell line, is commercially available through the ATCC. HepG2-A16 is a designated subclone from these cells. The study was conducted under the approved protocol, IM-04–08. “Research was conducted in compliance with the Animal Welfare Act and other federal statutes and regulations relating to animals and experiments involving animals and adheres to principles stated in the *Guide for the Care and Use of Laboratory Animals*, NRC Publication, 1996 edition. All procedures were reviewed and approved by the Institute's Animal Care and Use Committee, and performed in a facility accredited by the Association for Assessment and Accreditation of Laboratory Animal Care, International.”

### Malaria Antigens

A 522 bp DNA fragment of PfCelTOS 3D7 (Genbank Accession number AB194052) was designed by using the codon-harmonization algorithm [9]. The sequence was synthesized and provided in the PCR Blunt 3.5 kb vector (Retrogen, San Diego, CA). The PfCelTOS insert was subcloned into a modified pET(K−) expression vector (Novagen, Madison, WI), via a 5′ *Bam*HI and a 3′ *Not*I site, creating the pET(K−)PfCelTOS (3D7) plasmid. The resulting plasmid expressed PfCelTOS with an N-terminal six-histidine tag comprised within a 16 amino acid linker.

Briefly, recombinant PfCelTOS was expressed in B834 DE3 (F^−^ *ompT hsdS_B_* (r_B_^−^m_B_^−^) *gal dcm met* (DE3)) *E. coli* using phytone-based superbroth media containing 40 µg/ml kanamycin and 1.0% glucose. Protein was purified to homogeneity using two chromatographic steps; Ni^+2^-NTA Superflow (Qiagen) followed by Q anion exchanger (GE) (Fig.S1). The recombinant *P. berghei* CelTOS (PbCelTOS) used for stimulation of cellular responses was designed, synthesized, subcloned and expressed (BL21 DE3 (F^−^ *ompT hsdS_B_* (r_B_^−^m_B_^−^) *gal dcm* (DE3)) similar to PfCelTOS. PbCelTOS was purified to homogeneity using a two chromatographic step process; Ni^+2^-NTA Sepharose and Q anion exchanger. *E. coli* host cell protein contaminants (Cygnus Technology, *E. coli* HCP ELISA Kit) and residual endotoxin levels (Cape Cod Associates Pyrochrome LAL assay) were quantified for each antigen and found to be below the limits of detection.

### Immunizations

Mice used for immunizations were 6- to 8-week-old Balb/c-J (Jackson Laboratories, Bar Harbor ME) or CD-1 mice females from Charles River Laboratories (Wilmington MA). Mice were immunized subcutaneously in the scruff of the neck three times with 25 or 10 or 1 µg/dose of recombinant PfCelTOS or saline emulsified in Montanide ISA 720 (Seppic Fairfield NJ) [22].

### Challenge

Fourteen days after the final immunization, mice were challenged by subcutaneous inoculation (into the inguinal region) with 4,000 *P. berghei* sporozoites for Balb/c and 15,000 *P. berghei* sporozoites for CD-1 mice, dissected from infected mosquito salivary glands as described elsewhere [23]. The challenge dose was determined by titration studies in each mouse strain and compared to the different challenge routes [24]. Infection was determined by the presence of blood stage parasites in Giemsa stained thin blood smears on day 6 and day 8 after challenge [21]. Animals that were not infected at that time were tested again on day 14. Mice that remained un-infected by day 14 were classified as sterilely protected. We used this evaluation schedule because animals that are infected with *P. berghei* ANKA strain malaria parasites do not self-cure.

### ELISA

The ELISA was performed as previously described [21]. Briefly, 96-well plates (Immunolon 2 HB, Thermo Milford, MA) were coated by overnight incubation with recombinant PfCelTOS at a concentration of 0.5 µg/ml in PBS (100 µl/well) at 4°C. Plates were washed with PBS/0.1% Tween 20 using a 96-well plate automatic ELISA-plate washer (ScanWasher, Molecular Devices, Sunnyvale, CA) and blocked with blocking buffer (PBS, 1% BSA, pH 7.5) for 1 hr at 37°C. Sera were serially diluted in blocking buffer, incubated for 2 h at 37°C, and then washed. Alkaline phosphatase-conjugated goat anti-mouse IgG (Southern Biotechnology, Birmingham, AL) detection antibody was added in blocking buffer (1∶1,000) and incubated for 1 hr at RT. The assay was developed with BluePhos substrate (Kirkegaard Perry, Gaithersburg, MD) for 15 min at room temperature, then stopped with stopping solution (Kirkegaard Perry) and read at 570 nm (SpectraMax Plus^384^, Molecular Devices). Antibody concentration was determined by establishing a standard curve (run in parallel with each assay) with purified mouse IgG. For each serum tested, we determined a concentration that was within the linear portion of the reaction curve and used this dilution to extrapolate the actual antibody concentration in the assay wells.

### Immunofluorescence assay (IFA)

*P. falciparum* or *P. berghei* sporozoites were dissected from salivary glands of day 18 infected mosquitoes, then washed in Ozaki tubes and purified over DEAE columns as described elsewhere [23]. Eighteen-spot IFA slides (Cell Point, Gaithersburg, MD) were coated with approx. 4,000 sporozoites per spot, air-dried and then stored frozen at −30°C until analysis. Slides were thawed, blocked for 5 min with PBS with 1% BSA and then incubated with sera at various concentrations diluted in PBS with 1% BSA for 1 hr at RT. Spots were then washed with PBS and then incubated with goat-anti-mouse IgG-FITC (1∶100 dilution, Southern Biotech) for 45–60 min at RT. The slides were washed and mounted with Fluoromount mounting medium. All incubations were done in a humidified chamber followed by detection using fluorescence microscopy (Olympus BX41, 1,000 X magnification).

### Motility assays

IFA slides (Cell Point, Gaithersburg, MD) were pre-coated with RPMI-1640 (Invitrogen, Carlsbad, CA) containing 3% BSA (Sigma) for 15 min at 37°C. After removing the media, 10,000 sporozoites/well were added and incubated for 1 hr in a humidified chamber at 37°C. Slides and the deposited protein trails were fixed using 4% paraformaldehyde (10 min, RT). Spots were blocked with PBS containing 10% FBS for 45 min at 37°C and then incubated with anti-PfCSP mAb (216.5G10 or 8C10 or 2G3, PfCSP specific mAbs were a generous gift from Dr. Ted Hall, WRAIR) for 45 min at 37°C. Goat-anti-mouse-IgG-FITC was added (1∶100 dilution, Southern Biotech) to visualize the trails. Finally, slides were cover-slipped using Fluoromount mounting media and microscopy was performed (Olympus BX41, 1,000 X magnification). The number of sporozoites and trails per field were quantified and only trails were counted that were associated with sporozoites.

### Inhibition of Sporozoite Invasion assay (ISI)

A human hepatoma cell line, HepG2-A16, a subclone of HepG2 (ATCC, Manassas VA), was seeded at 60,000 cells per well on 8-well Glass LabTek Chamber slides (Thermo Fisher Scientific, Rochester NY) and allowed to adhere overnight. Sera were diluted and added to the hepatocytes to a final concentration of 1∶100 and 1∶400. *P. falciparum* CSP-specific mAb 49-1B2 (kindly provided by Dr. Ted Hall) was used as positive control, sera from adjuvant only immunized animals and complete media were used as negative controls. All experiment sera concentrations and controls were done in triplicate wells; 25,000 salivary gland sporozoites were added to each well and allowed to incubate for 3 hrs at 37°C in 5% CO_2_. Slides were washed with PBS and fixed with ice cold methanol for 10 minutes. Then they were washed with PBS and stained with the CSP-specific monoclonal antibody 49-1B2 (1∶10 dilution) as primary antibody. Goat-anti-mouse IgG-peroxidase (1∶200 dilution) (Kirkegaard Perry) was used as secondary antibody. Slides color was developed using the DAB Reagent Set (KPL Gaithersburg MD). Cover glasses were mounted with Permount (Science Company, Denver CO) and slides were evaluated with phase- contrast light microscopy (Olympus BX41, 200 X magnification).

### ELISpot

Multiscreen plates (Millipore, Bedford MA) were coated with either anti-IFN-γ or anti-IL-4 capturing antibodies according to manufacturer's instructions (R&D Systems, Minneapolis, MN). Plates were blocked using culture medium (DMEM containing 10% FBS, Pen/Strep, HEPES, NEAA, sodium pyruvate, 2-mercaptoethanol). Thawed splenocytes were counted and plated at 10^7^ cells/ml (50 µl/well). Recombinant proteins, either PbCelTOS or PfCelTOS, at these test concentrations, 30, 10, 3 µg/ml or 1 µg/ml of both anti-CD3 and anti-CD28 (as positive control) were used to stimulate the cultures. Plates were incubated for 36 hrs (IFN-γ) or 48 hrs (IL-4) and then processed according to manufacturer's instructions. Plates were counted using the AID Autoimmun Diagnostica GmbH ELISpot reader (Strassberg, Germany) and software.

### Monoclonal antibody production

Balb/c mice were immunized three times with 10 µg PfCelTOS emulsified in Montanide ISA 720 in three-week intervals. A fourth immunization was given three days prior to the fusion. The myeloma cell line SP-2 (ATCC Manassas, VA) was used as the fusion partner. Fusions were performed using 50% PEG1500 (Boehringer Mannheim, Germany) and hybridoma were selected using HAT media (Invitrogen).

### Statistical analysis

The protective effect of vaccination was evaluated using the Fisher's exact test comparing differences between the adjuvant alone control group and vaccine groups. Statistical significance of the serological data was tested using ANOVA analysis and Student T-tests (two-sided) employing the Minitab software package V14 (Penn State University, State College, PA).

## Supporting Information

Figure S1 Purification profile of recombinant PfCelTOS protein used for immunization and *in vitro* stimulations. Panel A Ni^+2^-NTA Sepharose, Lane 1: SeeBlue MW marker, Lane 2: *E. coli* lysate load, Lane 3: unbound flow-through, Lane 4: equilibration buffer wash, Lane 5: wash buffer 1, Lane 6: wash buffer 2, Lane 7: wash buffer 3, Lane 8: PfCelTOS elution from Ni^+2^-NTA resin. Panel B Q Sepharose, Lane 1: SeeBlue MW marker, Lane 2: Q load, Lane 3: Q flow through, Lane 4: equilibration buffer wash, Lane 5: Q wash buffer 1, Lane 6: Q wash buffer 2, Lane 7: Q elution. Final protein yield was 14 mg/g wet cells from this purification process. Residual host cell protein (HSP) content and endotoxin levels were determined as 0.4 ng HCP/50 µg dose and <0.6EU/mL, respectively, for the final product PfCelTOS.
(0.24 MB TIF)

Figure S2 CelTOS-specific antibodies impair motility of *P. falciparum* sporozoites. Sporozoites were pre-incubated with either control serum (Panel A) or a PfCSP-specific monoclonal antibody (Panel B) or PfCelTOS-specific antiserum (Panel C) at a final dilution of 1∶200. Images were taken at 1,000x magnification and are representative of 4 separate experiments.
(0.41 MB TIF)
