# Supplementary figures and images for: Immunization with Pre-Erythrocytic Antigen CelTOS from Plasmodium falciparum Elicits Cross-Species Protection against Heterologous Challenge with Plasmodium berghei
Source: PLoS One. 2010 Aug 19;5(8):e12294. doi: 10.1371/journal.pone.0012294 (PMC2924390; doi:10.1371/journal.pone.0012294)

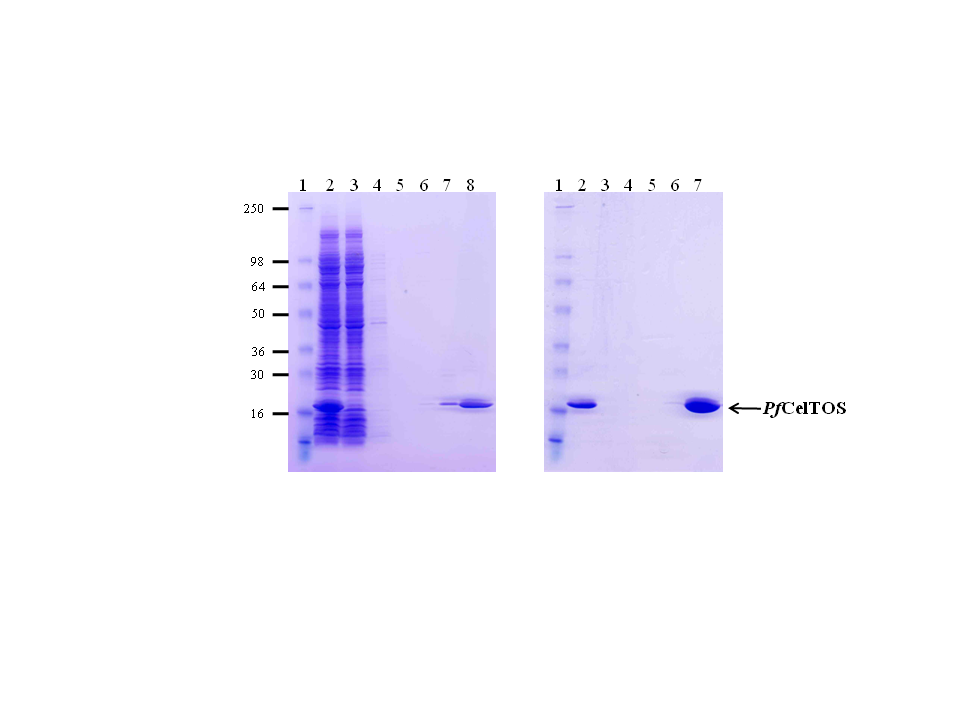

Supplement: Figure S1 — Purification profile of recombinant PfCelTOS protein used for immunization and in vitro stimulations. Panel A Ni+2-NTA Sepharose, Lane 1: SeeBlue MW marker, Lane 2: E. coli lysate load, Lane 3: unbound flow-through, Lane 4: equilibration buffer wash, Lane 5: wash buffer 1, Lane 6: wash buffer 2, Lane 7: wash buffer 3, Lane 8: PfCelTOS elution from Ni+2-NTA resin. Panel B Q Sepharose, Lane 1: SeeBlue MW marker, Lane 2: Q load, Lane 3: Q flow through, Lane 4: equilibration buffer wash, Lane 5: Q wash buffer 1, Lane 6: Q wash buffer 2, Lane 7: Q elution. Final protein yield was 14 mg/g wet cells from this purification process. Residual host cell protein (HSP) content and endotoxin levels were determined as 0.4 ng HCP/50 µg dose and <0.6EU/mL, respectively, for the final product PfCelTOS. (0.24 MB TIF) [file pone.0012294.s001.tif]

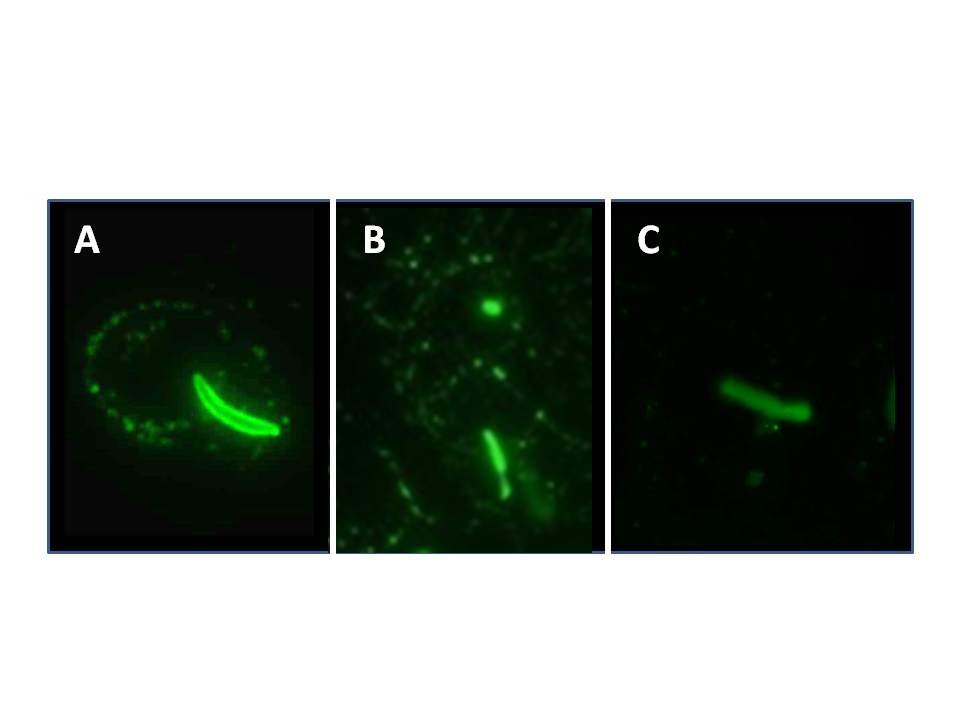

Supplement: Figure S2 — CelTOS-specific antibodies impair motility of P. falciparum sporozoites. Sporozoites were pre-incubated with either control serum (Panel A) or a PfCSP-specific monoclonal antibody (Panel B) or PfCelTOS-specific antiserum (Panel C) at a final dilution of 1∶200. Images were taken at 1,000x magnification and are representative of 4 separate experiments. (0.41 MB TIF) [file pone.0012294.s002.tif]
